# Intermediate and long-term residual cardiovascular risk in patients with established cardiovascular disease treated with statins

**DOI:** 10.3389/fcvm.2023.1308173

**Published:** 2024-01-15

**Authors:** K. Vijayaraghavan, S. Baum, N. R. Desai, S. J. Voyce

**Affiliations:** ^1^ Division of Cardiology, University of Arizona College of Medicine, Phoenix, AZ, United States; ^2^ Flourish Research, Boca Raton, FL, United States; ^3^ Charles E. Schmidt College of Medicine, Florida Atlantic University, Boca Raton, FL, United States; ^4^ Section of Cardiovascular Medicine, Department of Internal Medicine, Yale School of Medicine, New Haven, CT, United States; ^5^ Clinical Cardiology Research, Geisinger Heart Institute, Scranton, PA, United States

**Keywords:** secondary prevention, cardiovascular, long-term, eicosapentaenoic acid, omega-3 fatty acid, statins, residual risk

## Abstract

**Introduction:**

Statins remain the first-line treatment for secondary prevention of cardiovascular (CV) events, with lowering of low-density lipoprotein cholesterol (LDL-C) being their therapeutic target. Although LDL-C reduction significantly lowers CV risk, residual risk persists, even in patients with well-controlled LDL-C; thus, statin add-on agents that target pathways other than LDL-C, such as the omega-3 fatty acid eicosapentaenoic acid, may help to further reduce persistent CV risk in patients with established CV disease.

**Methods:**

This narrative review examines the contemporary literature assessing intermediate- and long-term event rates in patients with established CV disease treated with statins.

**Results:**

CV event rates among patients treated with statins who have established CV disease, including coronary artery disease, cerebrovascular disease, or peripheral arterial disease, accumulate over time, with a cumulative incidence of CV events reaching up to approximately 40% over 10 years. Recurrent stroke occurs in up to 19% of patients seven years after a first cerebrovascular event. Repeat revascularization and CV-related death occurs in up to 38% and 33% of patients with peripheral artery disease after three years, respectively.

**Discussion:**

Additional treatment strategies, such as eicosapentaenoic acid, are needed to reduce persistent CV risk in patients with established CV disease treated with statins.

## 1 Introduction

Globally, cardiovascular disease (CVD) remains the leading cause of morbidity and mortality and is a growing concern amidst an aging population with an increasingly sedentary lifestyle (1, 2). In 2019, nearly 18 million CVD-related deaths occurred (32% of global deaths), the majority (85%) of which were due to myocardial infarction (MI) and stroke (2). Individuals aged older than 75 years are three times more likely to die from CVD than younger individuals and will comprise more than 10% of the population by 2050 (3, 4).

In addition to the burden on patients, CVD places a substantial strain on the health care system in terms of resource utilization and costs. Between 2017 and 2018 in the United States, CVD accounted for $378 billion in direct and indirect costs, comprising 12% of total US health expenditures for that period and exceeding costs of any other major condition (5).

Patients with established atherosclerotic cardiovascular disease (ASCVD), including coronary artery disease (CAD), cerebrovascular artery disease, peripheral artery disease (PAD), or atherosclerotic aortic disease, are at very high risk for recurrent CVD events, including MI, stroke, and death (6). Approximately 20 million Americans aged 20 years or older have CAD (5). Approximately 20%–40% of acute coronary or cerebrovascular events occur in individuals with established vascular disease (7). The five-year CVD rate of MI, heart failure, stroke, or cardiovascular (CV)-related death in patients with established CVD is five-fold greater than that of individuals without CVD (7). As such, there is an unmet need to reduce the rate of CV events in this very-high-risk patient population.

In secondary prevention clinical practice, intermediate- and long-term rates of CV events are less known and less discussed than short-term outcomes after an acute event. Considering that there is a large patient population with established CVD, the focus should shift to longer-term outcomes based on published rates of events and death to facilitate patient-centered treatment decisions on additional preventive treatments that may lower residual risk beyond the effect of statins. The aim of this narrative review is to examine the contemporary literature assessing intermediate- and long-term event rates in patients with established CVD treated with statins.

## 2 Established link between low-density lipoprotein cholesterol, cardiovascular risk, and statin treatment

An elevated low-density lipoprotein cholesterol (LDL-C) level is a well-established risk factor for ASCVD and has been at the forefront of reducing CV risk in primary and secondary prevention settings (6, 8). Statins have remained first-line treatment for reducing CV risk for more than four decades, with overwhelming evidence showing a direct correlation between reduction of LDL-C levels and CV outcomes (8–10).

Early landmark statin efficacy studies, including the Scandinavian Simvastatin Survival Study (4S), Cholesterol and Recurrent Events trial, Long-Term Intervention With Pravastatin in Ischaemic Disease study (1998), and Heart Protection Study (2002), were pivotal in showing that statins are effective in reducing both LDL-C levels and CV events in patients with established CVD (11–14). Later landmark trials, including the Pravastatin or Atorvastatin Evaluation and Infection Therapy–Thrombolysis in Myocardial Infarction 22 and the Treating to New Targets study, compared the efficacy of high-intensity statins (80 mg) vs. standard-therapy statins (10–40 mg) and found that higher-intensity statin therapy further reduced CV events (15, 16).

Following the Pravastatin or Atorvastatin Evaluation and Infection Therapy–Thrombolysis in Myocardial Infarction 22 and the Treating to New Targets studies, a 2010 meta-analysis of 26 clinical trials involving 170,000 patients in primary and secondary prevention settings from the Cholesterol Treatment Trialists compared the efficacy of high- and low-intensity statin regimens and concluded that each 38.7-mg per dl reduction in LDL-C levels reduced the incidence of major vascular events by approximately 20% (17); these trials studied were instrumental in providing the evidence base for guidelines to recommend a target LDL-C level below 70 mg per dl (18).

Encouraged by results from the Pravastatin or Atorvastatin Evaluation and Infection Therapy–Thrombolysis in Myocardial Infarction 22 and the Treating to New Targets studies, efforts were focused on developing statin add-on agents that could lower LDL-C levels even further and reduce residual CV risk (19). Ezetimibe and proprotein convertase subtilisin/kexin type 9 (PCSK9) inhibitors (e.g., evolocumab, alirocumab) helped to reduce LDL-C levels to as low as 30 mg per dl when added to a statin and improved CV outcomes, as demonstrated in the Further Cardiovascular Outcomes Research With PCSK9 Inhibition in Subjects With Elevated Risk, Improved Reduction of Outcomes: Vytorin Efficacy International Trial (IMPROVE-IT) and Evaluation of Cardiovascular Outcomes After an Acute Coronary Syndrome During Treatment With Alirocumab (ODYSSEY OUTCOMES) trials, further adding to mounting evidence that a lower LDL-C level is better (19–22).

Guidelines have since been updated. The 2023 American Heart Association/American College of Cardiology guidelines for chronic coronary disease recommend high-intensity statin therapy for patients with a history of ASCVD to achieve at least 50% reduction in LDL-C (23). The 2023 American Diabetes Association “Standards of Care in Diabetes” recommend the addition of ezetimibe or a PCSK9 inhibitor if the LDL-C level remains at least 55 mg per dl in patients taking maximally tolerated statin therapy who are at very high risk (i.e., diabetes and atherosclerotic cardiovascular disease) (24). The 2019 European Society of Cardiology/European Atherosclerosis Society guidelines for dyslipidemia similarly recommend an LDL-C reduction of at least 50% from baseline and an LDL-C goal of less than 55 mg per dl for secondary prevention in patients at very high risk (25).

## 3 Despite significant advances in treatment for cardiovascular disease, residual risk persists, even in patients with low-density lipoprotein cholesterol levels below target level

Low-density lipoprotein cholesterol has remained the chief therapeutic target, including among statin add-on agents (e.g., ezetimibe, PCSK9 inhibitors, bempedoic acid), with the goal of reducing LDL-C levels further to lower residual risk (19–21, 26). Yet, a plethora of evidence indicates that substantial residual risk persists. One reason for this increased risk is suboptimal attainment of the guideline-recommended LDL-C level (i.e., <70 or 55 mg per dl) due, in part, to poor adherence to statin therapy (27, 28). Overall, 25%–50% of patients discontinue statin therapy within one year of their initiation, with high nonadherence even among those continuing treatment (29). However, even among patients with controlled LDL-C levels and those well below the recommended target of 70 or 55 mg per dl (19–21, 30–32), residual risk persists.

The following sections review CV risk in patients treated with statins who have established CVD, CAD, cerebrovascular disease, or PAD participating in intermediate and long-term registry and clinical studies published over the past decade.

## 4 Persistent cardiovascular risk in patients with established cardiovascular disease

The Reduction of Atherothrombosis for Continued Health Registry is a prospective cohort of 45,227 patients with established atherosclerosis (CAD, PAD, or cerebrovascular disease) or three or more risk factors for atherosclerosis and four years of follow-up. Cavender et al. (33) evaluated the impact of diabetes mellitus (DM) on long-term CV outcomes; statins were used at the four-year follow-up by 74.1% and 71.5% of patients with and without DM, respectively. The cumulative incidence of CV-related death, MI, or stroke in 19,699 patients with DM with known atherothrombosis varied from 5% to 7% at one year and increased to 14%–21% at approximately four years, depending on whether the patients had a prior ischemic event (Table 1) (33–38).

**Table 1:** Cumulative incidence of CV events in patients with established CVD treated with statins.

Study	Year	Country	Patient population	Follow-up, year	Outcomes	Time, %										
						1 year	2 years	3 years	4 years	5 years	6 years	7 years	8 years	9 years	10 years	11 years
Registry/cohort study																
REACH registry (33)	2015	44 countries	Established atherosclerosis and diabetes (*n* = 19,699)	4 (median)	CV-related death, MI, or stroke in patients with DM with known atherothrombosis	≈6	≈11	≈15	≈18 (3.8 years)	NA	NA	NA	NA	NA	NA	NA
					CV-related death, MI, or stroke in patients with DM with known atherothrombosis and prior ischemic event	≈7	≈13	≈17	≈21 (3.8 years)	NA	NA	NA	NA	NA	NA	NA
					CV-related death, MI, or stroke in patients with DM with known atherothrombosis and no prior ischemic event	≈5	≈8	≈12	≈14 (3.8 years)	NA	NA	NA	NA	NA	NA	NA
Clinical trial																
Heart Protection Study (35)	2011	United Kingdom	Increased risk of vascular events (*n* = 10,269)	11 (in trial and posttrial)	Nonfatal MI or coronary death, fatal or nonfatal stroke, coronary or noncoronary revascularization	≈5	≈10	≈16	≈20	≈25	≈28	≈32	≈36	≈39	≈42	≈44
FOURIER (20)	2017	49 countries	Established CVD and other risk factors (*n* = 13,780)		CV-related death, MI, stroke, hospitalization for unstable angina or coronary revascularization	6	11	15	NA	NA	NA	NA	NA	NA	NA	NA
					CV-related death, MI, or stroke	4	7	10	NA	NA	NA	NA	NA	NA	NA	NA
REDUCE-IT (38)	2019	11 countries	Established CVD or diabetes and other risk factors (*n* = 4,090)	4.9 (median)	CV-related death, nonfatal MI, nonfatal stroke, coronary revascularization, or unstable angina	≈5	≈11	≈16	≈21	≈25	NA	NA	NA	NA	NA	NA
					CV-related death, nonfatal MI, or nonfatal stroke	≈3	≈6	≈10	≈13	≈17	NA	NA	NA	NA	NA	NA
STRENGTH (34)	2020	22 countries	Established CVD or high-risk (*n* = 6,539)	3.5 (median)	CV-related death, nonfatal MI, nonfatal stroke, coronary revascularization, or unstable angina	≈4	≈7	≈10	≈13	NA	NA	NA	NA	NA	NA	NA
PROMINENT (37)	2022	24 countries	Patients with diabetes or without established CVD (*n* = 5,257)	3.4 (median)	MI, ischemic stroke, coronary revascularization, or death from CV-related causes	≈4	≈6	≈9	≈12	NA	NA	NA	NA	NA	NA	NA

CAD, coronary artery disease; CV, cardiovascular; CVD, cardiovascular disease; DM, diabetes mellitus; FOURIER, Further Cardiovascular Outcomes Research With PCSK9 Inhibition in Subjects With Elevated Risk; MI, myocardial infarction; NA, not available; PAD, peripheral artery disease; PROMINENT, Pemafibrate to Reduce Cardiovascular Outcomes by Reducing Triglycerides in Patients With Diabetes; REACH, Reduction of Atherothrombosis for Continued Health; REDUCE-IT, Reduction of Cardiovascular Events With Icosapent Ethyl-Intervention Trial; STRENGTH, Long-Term Outcomes Study to Assess Statin Residual Risk With Epanova in High Cardiovascular Risk Patients With Hypertriglyceridemia; TIA, transient ischemic attack.

The double-blind, randomized, multicenter Long-Term Outcomes Study to Assess Statin Residual Risk With Epanova in High Cardiovascular Risk Patients With Hypertriglyceridemia trial compared the effect of combined eicosapentaenoic acid (EPA) and docosahexaenoic acid (DHA) vs. placebo on clinical outcomes in 13,078 patients treated with statins at high CV risk (34). At randomization, all patients were receiving a statin, with 50% receiving high-intensity statins. At baseline, 56% (*n* = 6,539) of patients in the placebo group had established CVD, 46% had coronary disease, 8% had cerebrovascular disease, 4% had peripheral vascular disease, 4% had aortic disease, and 70% had DM. The median LDL-C level was 75 mg per dl, decreasing 1.1% in the placebo group during the study. The cumulative incidence of CV-related death, nonfatal MI, nonfatal stroke, coronary revascularization, or unstable angina among 6,539 patients receiving placebo was 4% at one year, increasing to 13% at four years in the placebo group (34).

Extended follow-up of the Heart Protection Study, which evaluated the efficacy and safety of considerably lowering LDL-C with simvastatin 40 mg in 20,536 patients at high risk for CV events, gathered long-term efficacy and safety data, including CV event rates, in the in-trial and posttrial periods totaling 11 years (35). Overall, 41% had MI, 24% had other history of CAD, 16% had cerebrovascular disease, and 33% had PAD (14). The baseline, in-trial, and posttrial LDL-C levels were 131.5, 89.0, and 100.5 mg per dl, respectively (35), and 85% and 74% of patients in the simvastatin group were taking a statin in-trial and posttrial, respectively (35). The cumulative incidence rates of nonfatal MI, coronary death, fatal/nonfatal stroke, or coronary/noncoronary revascularization in 10,269 patients receiving simvastatin were 5% at one year, 25% at five years, and 44% at 11 years (35).

The Further Cardiovascular Outcomes Research With PCSK9 Inhibition in Subjects With Elevated Risk trial, a multinational, double-blind, placebo-controlled study of 27,564 patients with established atherosclerosis treated with statins, investigated the efficacy of evolocumab vs. placebo for reducing CV-related death, MI, stroke, hospitalization for unstable angina or coronary (primary efficacy endpoint) and CV-related death, MI, or stroke (key secondary efficacy endpoint) (20). Nearly all patients were receiving either moderate- or high-intensity statin therapy, and the median LDL-C level at randomization was 92 mg per dl in the placebo group. The cumulative incidence of the primary endpoint in the 13,780 patients receiving placebo increased from 6% at one year to 15% at three years. The key secondary endpoint occurred in 4% at one year and in 10% cumulatively at three years.

The phase 3b, multicenter, randomized, double-blind, placebo-controlled Reduction of Cardiovascular Events With Icosapent Ethyl-Intervention Trial (REDUCE-IT) compared icosapent ethyl (IPE) 4 g with placebo in 8,179 patients with established CVD (secondary prevention) treated with statins or those with diabetes and other risk factors (primary prevention) (38). Eligible patients had a triglyceride (TG) level of 150–499 mg per dl and an LDL-C level of 41–100 mg per dl. Patients in the secondary prevention setting were aged 45 years or older with established CVD, and those in the primary prevention were aged 50 years or older with DM and had at least one additional risk factor. Overall, approximately 71% of the patient population had established CVD, and 58% had type 2 DM. Cumulative incidence of a composite of CV-related death, nonfatal MI, nonfatal stroke, coronary revascularization, or unstable angina in patients treated with statins receiving placebo in the trial was 5% at one year, increasing to 25% at five years. Cumulative incidence of CV-related death, nonfatal MI, or nonfatal stroke was 3% at one year and 17% at five years.

Pemafibrate to Reduce Cardiovascular Outcomes by Reducing Triglycerides in Patients With Diabetes (PROMINENT) was a multinational, double-blind, randomized, placebo-controlled trial involving 10,538 patients treated with statins (96%) who had type 2 DM, fasting TG levels 200–499 mg per dl, and high-density lipoprotein cholesterol levels no higher than 40 mg per dl who were randomized to receive pemafibrate 0.2 mg twice daily or matching placebo over a median of 3.4 years (37). Among 5,257 patients receiving placebo, approximately 67% had established CVD, 46% had type 2 DM for at least 10 years, 92% had hypertension, and 17% had a history of smoking. Overall, their LDL-C levels were well controlled, with mean baseline levels of 78 mg per dl. Among patients receiving placebo, the cumulative incidence of MI, ischemic stroke, coronary revascularization, or death from CV-related causes was 4% at one year, increasing to 12% at four years.

## 5 Residual cardiovascular risk in patients with established coronary artery disease

Using the French Registry of Acute Coronary Syndrome, Puymirat et al. (39) assessed the impact of an invasive strategy [i.e., early coronary angiography during initial hospital admission, whether it was followed (or not) by revascularization] compared with that of a conservative strategy (i.e., medical therapy alone with no coronary angiography) in 1,645 patients with non–ST-segment elevation MI admitted to an intensive care unit within 48 h after symptom onset for acute MI. Of 1,316 patients receiving invasive treatment, 34% were receiving a statin before admission and 76% were receiving a statin within 48 h of being admitted to the intensive care unit. In the invasive-treatment group, the cumulative incidence rates of overall mortality increased from 9% at one year to 17% at three years, CV-related death from 6% at one year to 8% at three years, nonfatal MI from 13% at one year to 22% at three years, and a composite of overall death, nonfatal MI, stroke, or revascularization from 21% at one year to 33% at three years (Table 2) (19, 21, 39–45).

**Table 2:** Cumulative incidence of CV events in patients with established CAD treated with statins.

Study	Year	Country	Patient population	Follow-up, year	Outcome	Time, %								
						1 year	2 years	3 years	4 years	5 years	6 years	7 years	10 years	16 years
Registry/cohort study														
FAST-MI (39)	2012	French Registry of Acute Coronary Syndrome	Acute MI receiving invasive strategy (*n* = 1,316)	3	Overall mortality	≈9	≈12	≈17	NA	NA	NA	NA	NA	NA
					CV mortality	≈6	≈7	≈8	NA	NA	NA	NA	NA	NA
					Nonfatal MI	≈12	≈16	≈22	NA	NA	NA	NA	NA	NA
					Overall mortality, nonfatal MI, stroke, or revascularization	≈21	≈27	≈33	NA	NA	NA	NA	NA	NA
Clinical trial														
CANTOS (40)	2017	39 countries	MI with hs-CRP level ≥2 mg/l (*n* = 3,344)	3.7 (median)	Nonfatal MI, nonfatal stroke, CV-related death	≈5	≈9	≈13	≈16	≈20	NA	NA	NA	NA
					Nonfatal MI, nonfatal stroke, CV-related death, or hospital stay for unstable angina that led to urgent revascularization	≈6	≈11	≈15	≈19	≈22	NA	NA	NA	NA
LIPID (41)	2016	87 centers in Australia and New Zealand	Acute MI or unstable angina pectoris (*n* = 4,512)	16	All-cause mortality	NA	≈2	NA	≈6	NA	≈11	NA	≈22	≈42
					CHD mortality	NA	≈2	NA	≈4	NA	≈6	NA	≈12	≈23
					CVD mortality	NA	≈2	NA	≈5	NA	≈7	NA	≈14	≈27
SEARCH (42)	2010	88 UK hospitals	MI (*n* = 6,031)	6.7 (mean)	Coronary death, MI, coronary revascularization	≈4	≈8	≈11	≈14	≈17	≈21	≈25	NA	NA
IMPROVE-IT (19)	2015	39 countries	ACS (*n* = 9,077)	6 (median)	Death from CVD, major coronary event, or nonfatal stroke	≈14	≈19	≈22	≈26	≈29	≈32	≈34	NA	NA
ALPS-AMI (43)	2015	20 sites in Japan	Acute MI (*n* = 253)	2	Death due to any cause, nonfatal MI, nonfatal stroke, congestive heart failure requiring hospital stay, or any type of coronary revascularization	≈24	≈31	NA	NA	NA	NA	NA	NA	NA
					Death due to any cause, nonfatal MI, or nonfatal stroke	≈1	≈4	NA	NA	NA	NA	NA	NA	NA
REAL-CAD (44)	2018	Japan	CAD (*n* = 6,199)	3.9 (median)	CV-related death, nonfatal MI, nonfatal ischemic stroke, or unstable angina requiring emergency hospital stay	≈1	≈2	≈4	≈5	NA	NA	NA	NA	NA
					CV-related death, nonfatal MI, nonfatal ischemic stroke, unstable angina requiring emergency hospital stay, or coronary revascularization based on clinical indication	≈3	≈5	≈7	≈9	NA	NA	NA	NA	NA
ODYSSEY OUTCOMES (21)	2018	57 countries	ACS (*n* = 9,462)	2.8 (median)	Death from CHD, nonfatal MI, fatal or nonfatal ischemic stroke, or unstable angina requiring hospital stay	≈5	≈9	≈12	≈15	NA	NA	NA	NA	NA
IMPROVE-IT (45)	2018	39 countries	ACS with (*n* = 6,604) or without DM (*n* = 2,459)	6 (median)	CV-related death, major coronary event, or nonfatal stroke in patients with DM	≈18	≈24	≈29	≈34	≈38	≈41	≈45	NA	NA
					CV-related death, major coronary event, or nonfatal stroke in patients without DM	≈12	≈17	≈20	≈24	≈26	≈28	≈31	NA	NA

ACS, acute coronary syndrome; ALPS-AMI, Assessment of Lipophilic Versus Hydrophilic Statin Therapy in Acute Myocardial Infarction; CAD, coronary artery disease; CANTOS, Canakinumab Antiinflammatory Thrombosis Outcome Study; CHD, coronary heart disease; CV, cardiovascular; CVD, cardiovascular disease; DM, diabetes mellitus; FAST-MI, French Registry of Acute Coronary Syndrome; FOURIER, Further Cardiovascular Outcomes Research With PCSK9 Inhibition in Subjects With Elevated Risk; hs-CRP, high-sensitivity C-reactive protein; IMPROVE-IT, Improved Reduction of Outcomes: Vytorin Efficacy International Trial; LIPID, Long-Term Intervention With Pravastatin in Ischaemic Disease; MI, myocardial infarction; NA, not available; REAL-CAD, Randomized Evaluation of Aggressive or Moderate Lipid Lowering Therapy With Pitavastatin in Coronary Artery Disease; SEARCH, Study of the Effectiveness of Additional Reductions in Cholesterol and Homocysteine.

The Canakinumab Antiinflammatory Thrombosis Outcome Study was a randomized, double-blind, placebo-controlled trial of 10,061 patients with a history of MI, and it evaluated the efficacy and safety of canakinumab for prevention of recurrent vascular events in patients with an hs-CRP level of at least 2 mg per l (40). Most patients had undergone a previous revascularization procedure, including percutaneous coronary intervention (PCI; 67%) and coronary artery bypass graft (14%). At baseline, of 3,344 patients in the placebo group, 91.1% were treated with statins, and the median LDL-C level was 82.4 mg per dl after 48 months of treatment. The cumulative incidence of nonfatal MI, nonfatal stroke, or CV-related death in the placebo group was 5% at the first year, increasing four-fold (20%) by the fifth year; the incidence of a composite of nonfatal MI, nonfatal stroke, CV-related death, or hospital stay for unstable angina that led to urgent revascularization was 6% at the first year, increasing to 22% by the fifth year (40).

Long-term effects of statin therapy were assessed in the Long-Term Intervention With Pravastatin in Ischaemic Disease study, which compared pravastatin 40 mg and placebo over six years in a double-blind phase and 10 years in an open-label phase, for a total of 16 years of follow-up data involving 9,014 patients with a history of MI or angina pectoris (41). Of the 4,512 patients in the original pravastatin group, 85% continued taking statin therapy during follow-up, and the median LDL-C level across the trial and follow-up period was 151 mg per dl. The cumulative incidences of all-cause mortality among patients taking pravastatin at six, 10, and 16 years were 11%, 22%, and 42%, respectively; of coronary heart disease mortality rates, 6%, 12%, and 23%, respectively; and, of CVD mortality rates, 7%, 14%, and 27%, respectively (41).

Investigators in the Study of the Effectiveness of Additional Reductions in Cholesterol and Homocysteine trial assessed the safety and efficacy of more intensive statin therapy with simvastatin 80 mg vs. 20 mg in 12,064 patients with a history of MI (42); 35% had previous coronary or noncoronary revascularization, 7% had cerebrovascular disease, 11% had DM, and 42% had hypertension. Among 6,031 participants receiving 80 mg simvastatin, 90% were compliant with treatment after 12 months and 77% after 84 months. By 84 months, LDL-C levels were reduced by approximately 14 mg per dl more in patients receiving simvastatin 80 mg vs. 20 mg. The cumulative incidence of coronary death, MI, or coronary revascularization in the simvastatin 80 mg group was 8% at two years and increased to 25% by seven years (42).

IMPROVE-IT, a double-blind, randomized trial involving patients admitted to the hospital for acute coronary syndrome (ACS), evaluated the effect of ezetimibe combined with simvastatin compared with simvastatin alone in 18,144 patients with a history of ACS (19). More than one-quarter (27%) of patients had DM, 88% had undergone coronary angiography, and 70% had undergone PCI. Overall, 34% were taking a statin drug at the time of the index event, and 77% received statin therapy during a hospital stay. The mean LDL-C level was approximately 94 mg per dl in both treatment groups at baseline and decreased to 69.5 mg per dl in the simvastatin monotherapy group over the course of the trial. The cumulative incidence of death from CVD, major coronary event, or nonfatal stroke in the 9,077 patients receiving simvastatin monotherapy was 14% at the first year and more than doubled (34%) by the seventh year (19).

Assessment of Lipophilic Versus Hydrophilic Statin Therapy in Acute MI was a multicenter, randomized trial comparing the efficacy of hydrophilic pravastatin with that of lipophilic atorvastatin in 528 patients with acute MI. Patients received either atorvastatin or pravastatin started at 10 mg once daily, with the goal of reducing LDL-C levels to below 100 mg per dl (43). Baseline mean LDL-C level was approximately 130 mg per dl in each treatment group; at the end of the study treatment (24 months), LDL-C levels were 92 and 82 mg per dl in the pravastatin and atorvastatin groups, respectively. The rate of death due to any cause, nonfatal MI, nonfatal stroke, congestive heart failure requiring hospital stay, or any type of coronary revascularization among 253 patients treated with pravastatin was 24% at the first year and increased to 31% by the second year. The rate of death due to any cause, nonfatal MI, or nonfatal stroke was 1% at the first year, increasing to 4% at the second year (43).

Randomized Evaluation of Aggressive or Moderate Lipid Lowering Therapy With Pitavastatin in Coronary Artery Disease was a prospective, multicenter, randomized, open-label, blinded end point study investigating the efficacy and safety of higher-dose (4 mg per day) vs. lower-dose (1 mg per day) pitavastatin in 13,054 Japanese patients with established CAD (44). Overall, 76% had hypertension, 40% had DM, 51% had prior MI, and 90% had prior coronary revascularization (predominantly by PCI). Baseline mean LDL-C levels were approximately 88 mg per dl in both treatment groups. At the end of the study treatment, LDL-C level was 76.6 mg per dl among the 6,199 patients in the high-dose pitavastatin group. The cumulative incidence of CV-related death, nonfatal MI, nonfatal ischemic stroke, or unstable angina requiring an emergency hospital stay was 1.2% at one year and increased to 4.6% at four years in the high pitavastatin group. Furthermore, in the same group, the incidence of CV-related death, nonfatal MI, nonfatal ischemic stroke, unstable angina requiring an emergency hospital stay, or coronary revascularization was 2.5% at one year and more than tripled to 8.5% at four years (44).

ODYSSEY OUTCOMES, a multicenter, randomized, double-blind, placebo-controlled trial involving 18,924 patients with ACS, evaluated the effect of alirocumab vs. placebo on risk of recurrent ischemic CV events (21). The majority of patients had MI (83%), and 16.8% had unstable angina. Of the 9,462 patients in the placebo group, 86.6% were receiving statin therapy at study end. Baseline mean LDL-C level was 92 mg per dl in both treatment groups, increasing to 103 mg per dl in the placebo group at the conclusion of the study. The cumulative incidence of death from coronary heart disease, nonfatal MI, fatal/nonfatal ischemic stroke, or unstable angina requiring a hospital stay was 5% at one year and increased to 15% at four years in the placebo group.

Subanalysis of the IMPROVE-IT trial investigated the efficacy and safety of ezetimibe plus simvastatin vs. placebo plus simvastatin in 18,135 patients with ACS stratified by the presence of DM at randomization (45). Patients with DM (*n* = 4,933) were more likely to have had prior MI or coronary artery bypass graft, less likely to present with ST-elevation MI, and more likely to have been treated with aspirin, beta blockers, statins, and/or angiotensin-converting enzyme inhibitors or angiotensin receptor blockers before the qualifying event than patients without DM. Overall, 29.8% and 46.9% of patients without or with DM were receiving a statin at baseline, respectively. The median LDL-C levels at baseline were 97 mg per dl in patients without DM and 89 mg per dl with DM; at study end, median LDL-C level was 65 mg per dl in the simvastatin plus placebo group. The cumulative incidence of CV-related death, major coronary event, or nonfatal stroke in the 2,459 patients without diabetes receiving simvastatin plus placebo was 12% at one year, increasing to 31% at seven years; the corresponding cumulative incidence in 6,604 patients with diabetes was 18% at one year, increasing to 45% at seven years.

## 6 Residual cardiovascular risk in patients with established cerebrovascular disease

Exploratory analysis of the Japan Statin Treatment Against Recurrent Stroke study, which initially evaluated the effect of pravastatin 10 mg on stroke recurrence in 1,095 patients with ischemic stroke, investigated the effect of on-treatment LDL-C and C-reactive protein levels on the risk of recurrent stroke and transient ischemic attack in patients with history of ischemic stroke (46). The mean baseline and on-treatment LDL-C levels were 118.9 and 101.7 mg per dl, respectively, for patients with LDL-C levels below 120 mg per dl (*n* = 591); mean baseline on-treatment LDL-C levels were 143.2 and 136.5 mg per dl, respectively, for patients with LDL-C levels of at least 120 mg per dl (*n* = 486). Overall, 65.7% of patients with LDL-C levels below 120 mg per dl and 30.5% of those with LDL-C at or above 120 mg per dl were taking a statin. The cumulative incidence of recurrent stroke and transient ischemic attack among patients with an LDL-C level of below 120 mg per dl was 3% at one year, increasing four-fold (12%) by six years (Table 3) (36, 46–48).

**Table 3:** Cumulative incidence of CV events in patients with established cerebrovascular disease treated with statins.

Study	Year	Country	Patient population	Follow-up, year	Outcome	Time, %							
						1 year	2 years	3 years	4 years	5 years	6 years	7 years	8 years
Registry/cohort study													
J-STARS (46)	2019	Japan	Noncardioembolic ischemic stroke with on-treatment LDL-C level <120 mg/dl (*n* = 591)	4.9	Recurrent stroke and TIA	≈3	≈5	≈7	≈9	≈10	≈12	NA	NA
Clinical trial													
IMPROVE-IT (47)	2017	39 countries	ACS and previous stroke (*n* = 346)	6 (median)	Stroke in patients with history of stroke	≈6	≈8	≈9	≈12	≈14	≈16	≈19	NA
TST (48)	2020	France and South Korea	Ischemic stroke with LDL-C < 70 mg/dl (*n* = 1,073) or LDL-C 90–110 mg/dl (*n* = 1,075)	5.3 (median)	Ischemic stroke, MI, new symptoms leading to urgent coronary or carotid revascularization, or death from CV causes (LDL-C < 70 mg/dl)	≈4	≈6	≈8	≈9	≈11	≈12	≈13	≈14
					Ischemic stroke, MI, new symptoms leading to urgent coronary or carotid revascularization, or death from CV causes (LDL-C 90–110 mg/dl)	≈6	≈7	≈10	≈12	≈14	≈16	≈17	≈18
FOURIER (36)	2020	49 countries	Established atherosclerosis with prior stroke (*n* = 2,651)	2.2 (median)	CV-related death, MI, stroke, hospital stay for unstable angina, or coronary revascularization	≈6	≈11	≈15 (2.9 years)	NA	NA	NA	NA	NA

ACS, acute coronary syndrome; CV, cardiovascular; FOURIER, Further Cardiovascular Outcomes Research With PCSK9 Inhibition in Subjects With Elevated Risk; IMPROVE-IT, Improved Reduction of Outcomes: Vytorin Efficacy International Trial; J-STARS, Japan Statin Treatment Against Recurrent Stroke; LDL-C, low-density lipoprotein cholesterol; MI, myocardial infarction; NA, not available; TIA, transient ischemic attack; TST, Treat Stroke to Target.

Subanalysis of IMPROVE-IT evaluated the efficacy of adding ezetimibe to simvastatin vs. simvastatin plus placebo for prevention of first and subsequent stroke and other CV events in patients with a history of stroke (47). Patients with prior stroke (*n* = 682) had DM (39%), had a current history of smoking (25%), and/or had hypertension (84%). The median LDL-C level at baseline for patients with prior stroke in the simvastatin group was 86 mg per dl, decreasing to 68 mg per dl at the 12-month follow-up period. The cumulative incidence of stroke in 346 patients with a history of stroke receiving simvastatin plus placebo was 6% at one year and more than tripled (19%) by seven years.

Exploratory analysis of a French cohort of the Treat Stroke to Target trial evaluated the benefit of targeting an LDL-C level below 70 mg per dl over 5.3 years to reduce the risk of CV events in 2,860 French and South Korean patients with ischemic stroke using the investigator's choice of statin (48). All patients were receiving statins throughout the trial. The cumulative incidences of ischemic stroke, MI, new symptoms leading to urgent coronary or carotid revascularization, or death from CV causes among 1,073 patients with LDL-C levels below 70 mg per dl were 6%, 11%, and 14% at two, five, and eight years, respectively; of the 1,075 patients with LDL-C levels of 90–110 mg per dl, the cumulative incidences of these same events were 7%, 14%, and 18% at two, five, and eight years, respectively.

Subanalysis of the Further Cardiovascular Outcomes Research With PCSK9 Inhibition in Subjects With Elevated Risk study investigated the efficacy of evolocumab vs. placebo for reducing CV events in a subset of statin-treated patients with prior stroke (36). Patients with a history of stroke before randomization had a higher risk profile than patients without prior stroke. Among 5,337 patients with prior stroke, 63% had ischemic stroke alone, 37% had polyvascular disease, and 21% had congestive heart failure. Nearly all patients (>99%) were taking a moderate- or high-intensity statin, and LDL-C level at baseline among patients with a history of stroke receiving placebo was 93 mg per dl and remained stable throughout follow-up. The cumulative incidence of CV-related death, MI, stroke, hospital stay for unstable angina, or coronary revascularization among the 2,651 patients with previous stroke receiving placebo was 6% at one year and increased to 15% at approximately three years of follow-up.

## 7 Residual cardiovascular risk in patients with established peripheral arterial disease

Kumbhani et al. (49) used the REACH registry, involving 69,055 patients with established coronary disease, cerebrovascular disease, or PAD, to investigate the association between statin use and limb outcomes in patients with PAD, defined as current intermittent claudication with an ankle-brachial index <0.9, history of intermittent claudication together with a previous intervention (e.g., angioplasty, stenting, atherectomy, peripheral arterial bypass graft), or both. A total of 5,861 patients had PAD; of these, nearly one-half (48.6%) had concomitant CAD, 22.4% had cerebrovascular disease, and 58.7% had polyvascular disease. Overall, 62.2% of patients were treated with statins; among these, 44.6% had DM, 94.3% had hypercholesterolemia, and 83.8% had hypertension. Among 3,643 patients taking statins, the cumulative incidence of adverse limb outcomes (defined as a composite of new PCI or surgical intervention, worsening claudication or new development of critical limb ischemia, or amputation) was 11% at a one-year follow-up and increased to 25% at approximately four years. The cumulative incidence of a composite of CV-related death, nonfatal MI, or nonfatal stroke was 5% at one year and increased to 20% at approximately four years (Table 4) (49–53).

**Table 4:** Cumulative incidence of CV events in patients with established peripheral vascular disease treated with statins (longitudinal studies).

Study	Year	Country	Patient population	Follow-up, year	Outcome	Time, %						
						1 year	2 years	3 years	4 years	5 years	6 years	7 years
REACH registry (49)	2014	44 countries	PAD (*n* = 3,643)	4	Adverse limb outcome		≈17	≈21	≈25 (3.75 years)	NA	NA	NA
					CV-related death/nonfatal MI/nonfatal stroke	≈5	≈11	≈16	≈20 (3.75 years)	NA	NA	NA
CRITISCH registry (50)	2017	27 German vascular centers	Critical limb ischemia receiving statins (*n* = 445)	2	Amputation	≈13	≈17	NA	NA	NA	NA	NA
					Total mortality	≈7	≈12	NA	NA	NA	NA	NA
Catalan primary care (51)	2016	Spanish primary care database	Low ankle brachial index (*n* = 2,740)	3.6 (median)	Major CV event	≈2	≈4	≈6	≈8	≈10	≈11	≈12
					Total mortality	≈2	≈4	≈7	≈10	≈13	≈16	≈20
Single-center cohort (53)	2018	Switzerland	PAD (*n* = 769)	4.2 (median)	CV mortality	≈2	≈3	≈4	≈6	≈7	≈8	≈7 (7.7 years)
Multicenter CV database (52)	2013	11 Japanese vascular surgery centers	Critical limb ischemia (*n* = 169)	4	Repeat revascularization	≈28	≈38	≈38	≈38	NA	NA	NA
					CV mortality	≈8	≈12	≈15	≈15	NA	NA	NA
					Total mortality	≈18	≈26	≈29	≈33	NA	NA	NA

CRITISCH, First-Line Treatments in Patients With Critical Limb Ischemia; CV, cardiovascular; MI, myocardial infarction; NA, not available; PAD, peripheral artery disease; REACH, Reduction of Atherothrombosis for Continued Health.

Stavroulakis et al. (50) used the CRITISCH registry, a prospective multicenter registry of 1,200 patients with critical limb ischemia, to investigate the impact of statin therapy on outcomes of patients with critical limb ischemia. Among 445 patients receiving statins, the cumulative incidence rates of amputation were 13% and 17% at the one- and two-year follow-ups, respectively, and the total mortality rates were 7% at one year and 12% at two years.

Using the Catalan Primary Care System's Clinical Records Database, Ramos et al. (51) evaluated whether statin use reduced the incidence of CVD and death among 5,480 patients with an ankle-brachial index at or below 0.95 and without clinically recognized CVD. The mean baseline LDL-C level was 132.5 mg per dl, and 95.8% were taking a moderate- or high-intensity statin. The cumulative incidence of major adverse cardiac events—a composite of hard coronary heart disease (MI, cardiac revascularization, or coronary death) and stroke (fatal/nonfatal ischemic stroke)—among the 2,740 patients receiving statins was 2% at one year and increased to 12% at seven years of follow-up. The cumulative incidence of all-cause mortality was 2% at one year and increased to 20% at seven years.

In the Japanese Below-the-Knee Artery Treatment II trial multicenter registry, Tomoi et al. (52) evaluated the efficacy of statin treatment after endovascular treatment in 812 patients with critical limb ischemia due to isolated below-the-knee lesions. Of the 169 patients receiving statin treatment, 74% had hypertension, 71% had dyslipidemia, 80.5% had DM, 46.7% had chronic kidney disease, 19.5% had cerebrovascular disease, and 52.7% had CAD. The mean baseline LDL-C in patients receiving statins was 95 mg per dl. The cumulative incidence of repeat revascularization among patients receiving statins was 28% at one year and increased to approximately 38% at two to four years. In addition, the cumulative incidence of all-cause mortality was 26% at two years and increased to 33% at four years. The CV mortality rate was 12% and increased to 15% at four years.

In a large observational study, Dopheide et al. (53) sought to assess trends in LDL-C target level attainment and CV mortality rates over time between the years 2010 and 2017 among 1,109 patients with symptomatic PAD undergoing lower-limb revascularization. The majority of patients had hypertension (86.2%), 44.2% had CAD, and 17.5% had other CVD. The prevalence of CV risk factors in the study population was high, with nearly two-thirds with a current history of smoking and one-third with DM. Overall, 769 (69%) patients were receiving statins, but achievement of the LDL-C target level remained poor. Most (>70%) patients in the overall study population did not achieve an LDL-C level below 70 mg per dl. However, LDL-C levels did improve over time, decreasing from an average of 110 mg per dl in 2010 to 80 mg per dl in 2017. Among patients taking statins, the cumulative incidence of CV mortality was 3% at two years, increasing to 8% by seven years.

## 8 Discussion

Intermediate- and long-term studies of patients receiving statins with established ASCVD, CAD, cerebrovascular disease, or PAD demonstrate that substantial, persistent CV risk exists over time. Of the six studies involving patients with any type of established ASCVD, the highest reported cumulative incidence of events (nonfatal MI or coronary death, fatal/nonfatal stroke, coronary/noncoronary revascularization) was 44% by 11 years (35). Similarly, among the nine studies of patients with established CAD, the highest reported cumulative incidence of events (CV-related death, major coronary event, or nonfatal stroke) was 45% by seven years in patients with ACS and DM (45). Of the four studies involving patients with established cerebrovascular disease, the highest reported cumulative incidence of recurrent stroke was 19% at seven years (47). Among the five studies involving patients with established PAD, the highest reported cumulative incidence rates of repeat revascularization and CV-related death were 38% and 33% at three years, respectively, in patients with critical limb ischemia.

Cardiovascular prevention efforts have historically focused on lowering LDL-C levels, with statins, PCSK9 inhibitors, ezetimibe, and bempedoic acid all targeting LDL-C as their primary mechanism of action (26). However, CV risk persists in patients with well-controlled LDL-C levels (19–22, 30–32), suggesting that sources other than LDL-C contribute to residual risk and that research efforts should focus on statin add-on agents with mechanisms of action beyond LDL-C lowering (32, 54).

The source of this residual CV risk is multifactorial and includes lipid and nonlipid factors (32), such as smoking, obesity, DM, elevated TG levels or low high-density lipoprotein cholesterol, non–high-density lipoprotein cholesterol, apolipoprotein B levels, lipoprotein (a) [Lp(a)], and LDL particle numbers (32, 55). Hypertriglyceridemia (TG level ≥150 mg/dl) is a common condition, with an estimated prevalence of 31% in the adult US population (56). Epidemiologic, clinical, and genetic studies, including Mendelian randomization studies, support the independent role of elevated TG levels in signaling increased ASCVD risk (57–59).

Triglyceride-lowering drugs, including fibrates, niacin, and omega-3 fatty acids, both mixed (containing both EPA and DHA) and EPA-alone formulations, have been extensively studied for lowering CV risk (34, 38, 60–68). However, fibrates, niacin, and mixed omega-3 fatty acids have not yielded consistent CV benefits (34, 60–63). Conversely, EPA-alone formulations, including the highly purified ethyl ester IPE, markedly improved CV outcomes in patients treated with statins (38, 64–68). Unlike other approved statin add-on agents, the primary mechanism of action of IPE for reducing CV risk is not related to further lowering of LDL-C levels (69); rather, serum EPA levels are likely the primary driver of reduction in CV events and related to its pleiotropic mechanisms of action, largely comprising nonlipid effects, including effects on foam cell formation, inflammation, plaque formation/progression, platelet aggregation, and plaque rupture (69). In an early study by Bang and Dyerberg, Inuit living in West Greenland had lower serum levels of cholesterol, apolipoprotein B, and triglycerides and lower rates of coronary heart disease compared with Inuit living in Denmark but had prolonged bleeding times. The authors suggested that the high dietary intake of EPA and the low intake of linoleic acid in the Greenlandic Inuit were responsible for delayed onset of atherosclerosis and a shift in the balance between pro- and antiaggregatory prostaglandins toward antiaggregation, resulting in a low rate of death due to CV disease (70, 71). A similar early study by Hirai et al. (72) compared plasma levels of EPA from Japanese inhabitants of a fishing village who consumed an average of 250 g of fish daily compared with those from inhabitants of a farming village who consumed an average of 90 g fish daily. Plasma levels of EPA were significantly higher among those living in the fishing village than in the farming village. Platelet aggregation studies were carried out to determine the concentration of adenosine diphosphate (ADP) in platelet-rich plasma that would induce a >50% maximum aggregation; the ADP level producing 50% maximum aggregation was higher in the people of the fishing village than in those of the farming village. The authors concluded that hemostatic function could be manipulated with the ingestion of a fish-rich diet with potential beneficial effects on CV disorders through the reduction of platelet aggregability.

Initially approved in 2012 for hypertriglyceridemia, IPE received a second indication in 2019 as an adjunct to maximally tolerated statin therapy to reduce the risk of MI, stroke, coronary revascularization, and unstable angina requiring hospital stays in adult patients with an elevated TG level (≥150 mg per dl) and established CVD or DM and at least two additional risk factors for CVD (73). This approval was based on results from the pivotal REDUCE-IT, in which IPE 4 g per day reduced the primary endpoint (i.e., composite of CV-related death, nonfatal MI, nonfatal stroke, coronary revascularization, or unstable angina) by 25% and the secondary endpoint (i.e., composite of CV-related death, nonfatal MI, or nonfatal stroke) by 26% vs. placebo in patients treated with statins (both *p *< 0.001) (38).

Other randomized trials of purified EPA have similarly shown benefit in CV outcomes. The Japan EPA Lipid Intervention Study, an open-label trial involving 18,645 patients receiving EPA 1.8 g per day plus a statin or a statin alone, showed a 19% reduction in CV events with EPA plus a statin vs. statin monotherapy (64). Nosaka et al. (67) performed a prospective, randomized, open-label trial involving 238 patients with ACS treated with PCI. They reported a 58% reduction in CV events among patients receiving purified EPA 1.8 g per day with a statin compared with statin monotherapy after one year of follow-up (*p *= 0.02). A recent trial, the Randomized Trial for Evaluation in Secondary Prevention Efficacy of Combination Therapy - Statin and EPA, involved 2,460 Japanese patients aged 20–79 years with long-term CAD and a low EPA-to-arachidonic acid ratio (<0.4) who were treated with statins. Patients were randomized into two groups: one group received purified EPA 1.8 g per day in addition to statin therapy (*n* = 1,225), while the other group received statin therapy alone (*n* = 1,235). The purified EPA group had a clinically significant reduction of 21.5% in CV risk in the primary endpoint (*p *= 0.05) and a significant reduction of 26.6% in the secondary composite endpoint compared to the statin only group (*p *= 0.03). EPA concentrations increased from 48.5 at baseline to 140.5 µg per dl after three years of follow-up in the EPA group, while the statin monotherapy group had a baseline concentration of 46.6 and 51.5 µg per dl at follow-up (*p *< 0.05 between groups) (68). Imaging findings from the Combination Therapy of Eicosapentaenoic Acid and Pitavastatin for Coronary Plaque Regression Evaluated by Integrated Backscatter Intravascular Ultrasonography and the Effect of Vascepa on Improving Coronary Atherosclerosis in People With High Triglycerides Taking Statin Therapy studies provide further evidence of the benefit of purified EPA on CV outcomes, including significant reduction in coronary plaque volume (65, 66).

The significant benefit of IPE in REDUCE-IT has been criticized by some who attributed the encouraging data with IPE to the negative effects of mineral oil placebo in increased LDL-C and hs-CRP levels (74), even after the US Food and Drug Administration concluded that increases in LDL-C and hs-CRP levels are likely to have had negligible effects on results from REDUCE-IT (75, 76). Furthermore, recent results from the Randomized Trial for Evaluation in Secondary Prevention Efficacy of Combination Therapy - Statin and EPA add to existing evidence from randomized CV outcome trials [Japan EPA Lipid Intervention Study and Nosaka et al. (67) trials] that the significant reductions in CV events reported in REDUCE-IT are valid, as all three studies did not include a mineral oil placebo but achieved significant reductions in CV events comparable with, if not better than, those seen in REDUCE-IT (38, 64, 67, 68). Furthermore, the trials in Japanese populations show that even among patients with high serum concentrations of EPA due to heavy fish consumption, concomitant treatment with statins and EPA is beneficial in reducing CV risk.

Since publication of REDUCE-IT, widespread recognition of the impact of IPE in reducing CV events has been reflected in numerous US and international guidelines, many of which recommend use of IPE for CV risk reduction (77–80).

Outside the lipid-specific space, there are other secondary prevention therapies aimed at reducing residual CV risk that target thrombosis and inflammation (81). In patients with stable ASCVD, adding low-dose rivaroxaban (2.5 mg twice daily) to aspirin was associated with a 24% relative risk reduction in a composite of CV death, stroke, or MI compared with aspirin alone (82) and should be considered in patients with CAD at high risk of ischemic events without a high bleeding risk (83, 84). Among patients with comorbid DM and ASCVD, glucagon-like 1 receptor agonists (GLP1-RAs) and sodium glucose cotransporter 2 (SGLT2) inhibitors are recommended to improve CVD and cardiorenal outcomes (83, 84). Previous studies on the use of SGLT2 inhibitors and GLP1-RA have shown a decreased incidence of MI, stroke, and CV death by 12%–14% in patients with ASCVD (85, 86).

Research on lipoprotein(a) [Lp(a)] lowering therapies is of interest in the context of potential therapies to reduce residual risk in patients with established CVD. Lp(a) has been identified as a causal risk factor for ASCVD leading to atherosclerosis, thrombosis, and inflammation. Currently, lipid-lowering medications have shown only modest Lp(a) lowering and/or unknown CV outcome benefits; however, several therapies targeting Lp(a) are currently in clinical development (87) and are being evaluated for their impact on CV outcomes (88).

## 9 Conclusion

This review of intermediate and long-term studies involving patients with a history of CVD receiving statins demonstrated that significant risk persists and CV events may occur in up to approximately 40% of patients over 10 years. As such, efforts have focused on the development of statin add-on agents to further reduce CV risk through pathways other than lowering of LDL-C levels. Additional treatment strategies, such as EPA, are needed to reduce persistent CV risk in patients with established CV disease treated with statins.

## References

[1] Park JH Moon JH Kim HJ Kong MH Oh YH. Sedentary lifestyle: overview of updated evidence of potential health risks. Korean J Fam Med. (2020) 41(6):365–73. 10.4082/kjfm.20.0165 33242381 PMC7700832

[2] World Health Organization. Cardiovascular diseases (CVDs) (2021). Available online at: https://www.who.int/news-room/fact-sheets/detail/cardiovascular-diseases-(cvds) (Accessed December 20, 2023).

[3] World Population Ageing. New York: United Nations, Department of Economic and Social Affairs, Population Division (2015).

[4] Cobos-Palacios L Sanz-Cánovas J Muñoz-Ubeda M Lopez-Carmona MD Perez-Belmonte LM Lopez-Sampalo A Statin therapy in very old patients: lights and shadows. Front Cardiovasc Med. (2021) 8:779044. 10.3389/fcvm.2021.779044 34912868 PMC8667269

[5] Tsao CW Aday AW Almarzooq ZI Alonso A Beaton AZ Bittencourt MS Heart disease and stroke statistics-2022 update: a report from the American Heart Association. Circulation. (2022) 145(8):e153–639. 10.1161/cir.0000000000001052 35078371

[6] Grundy SM Stone NJ Bailey AL Beam C Birtcher KK Blumenthal RS 2018 AHA/ACC/AACVPR/AAPA/ABC/ACPM/ADA/AGS/APhA/ASPC/NLA/PCNA guideline on the management of blood cholesterol: a report of the American College of Cardiology/American Heart Association Task Force on Clinical Practice Guidelines. J Am Coll Cardiol. (2019) 73(24):e285–350. 10.1016/j.jacc.2018.11.003 30423393

[7] World Heart Federation. Roadmap for secondary prevention of cardiovascular disease (2015). Available online at: https://world-heart-federation.org/cvd-roadmaps/whf-global-roadmaps/secondary-prevention/ (Accessed December 20, 2023).

[8] Ference BA Ginsberg HN Graham I Ray KK Packard CJ Bruckert E Low-density lipoproteins cause atherosclerotic cardiovascular disease. 1. Evidence from genetic, epidemiologic, and clinical studies. A consensus statement from the European Atherosclerosis Society Consensus Panel. Eur Heart J. (2017) 38(32):2459–72. 10.1093/eurheartj/ehx144 28444290 PMC5837225

[9] Morofuji Y Nakagawa S Ujifuku K Fujimoto T Otsuka K Niwa M Beyond lipid-lowering: effects of statins on cardiovascular and cerebrovascular diseases and cancer. Pharmaceuticals. (2022) 15(2):151. 10.3390/ph15020151 35215263 PMC8877351

[10] Harrington RA. Statins-almost 30 years of use in the United States and still not quite there. JAMA Cardiol. (2017) 2(1):66. 10.1001/jamacardio.2016.4709 27842172

[11] Scandinavian Simvastatin Survival Study Group. Randomised trial of cholesterol lowering in 4444 patients with coronary heart disease: the Scandinavian Simvastatin Survival Study (4S). Lancet. (1994) 344(8934):1383–9. 10.1016/S0140-6736(94)90566-5 7968073

[12] Sacks FM Pfeffer MA Moye LA Rouleau JL Rutherford JD Cole TG The effect of pravastatin on coronary events after myocardial infarction in patients with average cholesterol levels. N Engl J Med. (1996) 335(14):1001–9. 10.1056/NEJM199610033351401 8801446

[13] LIPID Study Group. Long-term effectiveness and safety of pravastatin in 9014 patients with coronary heart disease and average cholesterol concentrations: the LIPID trial follow-up. Lancet. (2002) 359(9315):1379–87. 10.1016/s0140-6736(02)08351-4 11978335

[14] Heart Protection Study Collaborative Group. MRC/BHF heart protection study of cholesterol lowering with simvastatin in 20,536 high-risk individuals: a randomised placebo-controlled trial. Lancet. (2002) 360(9326):7–22. 10.1016/S0140-6736(02)09327-3 12114036

[15] LaRosa JC Grundy SM Waters DD Shear C Barter P Fruchart JC Intensive lipid lowering with atorvastatin in patients with stable coronary disease. N Engl J Med. (2005) 352(14):1425–35. 10.1056/NEJMoa050461 15755765

[16] Cannon CP Braunwald E McCabe CH Rader DJ Rouleau JL Belder R Intensive versus moderate lipid lowering with statins after acute coronary syndromes. N Engl J Med. (2004) 350(15):1495–504. 10.1056/NEJMoa040583 15007110

[17] Baigent C Blackwell L Emberson J Holland LE Reith C Bhala N Efficacy and safety of more intensive lowering of LDL cholesterol: a meta-analysis of data from 170,000 participants in 26 randomised trials. Lancet. (2010) 376(9753):1670–81. 10.1016/S0140-6736(10)61350-5 21067804 PMC2988224

[18] Leibowitz M Cohen-Stavi C Basu S Balicer RD. Targeting LDL cholesterol: beyond absolute goals toward personalized risk. Curr Cardiol Rep. (2017) 19(6):52. 10.1007/s11886-017-0858-6 28432662 PMC5815375

[19] Cannon CP Blazing MA Giugliano RP McCagg A White JA Theroux P Ezetimibe added to statin therapy after acute coronary syndromes. N Engl J Med. (2015) 372(25):2387–97. 10.1056/NEJMoa1410489 26039521

[20] Sabatine MS Giugliano RP Keech AC Honarpour N Wiviott SD Murphy SA Evolocumab and clinical outcomes in patients with cardiovascular disease. N Engl J Med. (2017) 376(18):1713–22. 10.1056/NEJMoa1615664 28304224

[21] Schwartz GG Steg PG Szarek M Bhatt DL Bittner VA Diaz R Alirocumab and cardiovascular outcomes after acute coronary syndrome. N Engl J Med. (2018) 379(22):2097–107. 10.1056/NEJMoa1801174 30403574

[22] Bohula EA Giugliano RP Cannon CP Zhou J Murphy SA White JA Achievement of dual low-density lipoprotein cholesterol and high-sensitivity C-reactive protein targets more frequent with the addition of ezetimibe to simvastatin and associated with better outcomes in IMPROVE-IT. Circulation. (2015) 132(13):1224–33. 10.1161/circulationaha.115.018381 26330412

[23] Virani SS Newby LK Arnold SV Bittner V Brewer LC Demeter SH 2023 AHA/ACC/ACCP/ASPC/NLA/PCNA guideline for the management of patients with chronic coronary disease: a report of the American Heart Association/American College of Cardiology Joint Committee on Clinical Practice Guidelines. J Am Coll Cardiol. (2023) 82(9):833–955. 10.1016/j.jacc.2023.04.003 37480922

[24] ElSayed NA Aleppo G Aroda VR Bannuru RR Brown FM Bruemmer D Cardiovascular disease and risk management: standards of care in diabetes-2023. Diabetes Care. (2023) 46(suppl 1):S158–90. 10.2337/dc23-S010 36507632 PMC9810475

[25] Mach F Baigent C Catapano AL Koskinas KC Casula M Badimon L 2019 ESC/EAS guidelines for the management of dyslipidaemias: lipid modification to reduce cardiovascular risk: the task force for the management of dyslipidaemias of the European Society of Cardiology (ESC) and European Atherosclerosis Society (EAS). Eur Heart J. (2020) 41(1):111–88. 10.1093/eurheartj/ehz455 31504418

[26] Pirillo A Catapano AL. New insights into the role of bempedoic acid and ezetimibe in the treatment of hypercholesterolemia. Curr Opin Endocrinol Diabetes Obes. (2022) 29(2):161–6. 10.1097/med.0000000000000706 34980867 PMC8915986

[27] Colantonio LD Rosenson RS Deng L Monda KL Dai Y Farkouh ME Adherence to statin therapy among US adults between 2007 and 2014. J Am Heart Assoc. (2019) 8(1):e010376. 10.1161/jaha.118.010376 30616455 PMC6405715

[28] Wang Q Liang C. Role of lipid-lowering therapy in low-density lipoprotein cholesterol goal attainment: focus on patients with acute coronary syndrome. J Cardiovasc Pharmacol. (2020) 76(6):658–70. 10.1097/fjc.0000000000000914 33002965 PMC7720869

[29] Brown MT Bussell JK. Medication adherence: WHO cares? Mayo Clin Proc. (2011) 86(4):304–14. 10.4065/mcp.2010.0575 21389250 PMC3068890

[30] Kaasenbrood L Boekholdt SM van der Graaf Y Ray KK Peters RJ Kastelein JJ Distribution of estimated 10-year risk of recurrent vascular events and residual risk in a secondary prevention population. Circulation. (2016) 134(19):1419–29. 10.1161/circulationaha.116.021314 27682883

[31] Gynnild MN Hageman SHJ Dorresteijn JAN Spigset O Lydersen S Wethal T Risk stratification in patients with ischemic stroke and residual cardiovascular risk with current secondary prevention. Clin Epidemiol. (2021) 13:813–23. 10.2147/clep.s322779 34566434 PMC8456548

[32] Baum SJ Scholz KP. Rounding the corner on residual risk: implications of REDUCE-IT for omega-3 polyunsaturated fatty acids treatment in secondary prevention of atherosclerotic cardiovascular disease. Clin Cardiol. (2019) 42(9):829–38. 10.1002/clc.23220 31254481 PMC6727875

[33] Cavender MA Steg PG Smith SC Jr Eagle K Ohman EM Goto S Impact of diabetes mellitus on hospitalization for heart failure, cardiovascular events, and death: outcomes at 4 years from the Reduction of Atherothrombosis for Continued Health (REACH) Registry. Circulation. (2015) 132(10):923–31. 10.1161/circulationaha.114.014796 26152709

[34] Nicholls SJ Lincoff AM Garcia M Bash D Ballantyne CM Barter PJ Effect of high-dose omega-3 fatty acids vs corn oil on major adverse cardiovascular events in patients at high cardiovascular risk: the STRENGTH randomized clinical trial. JAMA. (2020) 324(22):2268–80. 10.1001/jama.2020.22258 33190147 PMC7667577

[35] Heart Protection Study Collaborative Group. Effects on 11-year mortality and morbidity of lowering LDL cholesterol with simvastatin for about 5 years in 20,536 high-risk individuals: a randomised controlled trial. Lancet. (2011) 378(9808):2013–20. 10.1016/s0140-6736(11)61125-2 22115874 PMC3242163

[36] Giugliano RP Pedersen TR Saver JL Sever PS Keech AC Bohula EA Stroke prevention with the PCSK9 (proprotein convertase subtilisin-kexin type 9) inhibitor evolocumab added to statin in high-risk patients with stable atherosclerosis. Stroke. (2020) 51(5):1546–54. 10.1161/strokeaha.119.027759 32312223

[37] Das Pradhan A Glynn RJ Fruchart JC MacFadyen JG Zaharris ES Everett BM Triglyceride lowering with pemafibrate to reduce cardiovascular risk. N Engl J Med. (2022) 387(21):1923–34. 10.1056/NEJMoa2210645 36342113

[38] Bhatt DL Steg PG Miller M Brinton EA Jacobson TA Ketchum SB Cardiovascular risk reduction with icosapent ethyl for hypertriglyceridemia. N Engl J Med. (2019) 380(1):11–22. 10.1056/NEJMoa1812792 30415628

[39] Puymirat E Taldir G Aissaoui N Lemesle G Lorgis L Cuisset T Use of invasive strategy in non-ST-segment elevation myocardial infarction is a major determinant of improved long-term survival: FAST-MI (French Registry of Acute Coronary Syndrome). JACC Cardiovasc Interv. (2012) 5(9):893–902. 10.1016/j.jcin.2012.05.008 22995875

[40] Ridker PM Everett BM Thuren T MacFadyen JG Chang WH Ballantyne C Antiinflammatory therapy with canakinumab for atherosclerotic disease. N Engl J Med. (2017) 377(12):1119–31. 10.1056/NEJMoa1707914 28845751

[41] Hague WE Simes J Kirby A Keech AC White HD Hunt D Long-term effectiveness and safety of pravastatin in patients with coronary heart disease: sixteen years of follow-up of the LIPID study. Circulation. (2016) 133(19):1851–60. 10.1161/circulationaha.115.018580 27016105

[42] Armitage J Bowman L Wallendszus K Bulbulia R Rahimi K Haynes R Intensive lowering of LDL cholesterol with 80 mg versus 20 mg simvastatin daily in 12,064 survivors of myocardial infarction: a double-blind randomised trial. Lancet. (2010) 376(9753):1658–69. 10.1016/s0140-6736(10)60310-8 21067805 PMC2988223

[43] Izawa A Kashima Y Miura T Ebisawa S Kitabayashi H Yamamoto H Assessment of lipophilic vs. hydrophilic statin therapy in acute myocardial infarction – ALPS-AMI study. Circ J. (2015) 79(1):161–8. 10.1253/circj.CJ-14-0877 25392071

[44] Taguchi I Iimuro S Iwata H Takashima H Abe M Amiya E High-dose versus low-dose pitavastatin in Japanese patients with stable coronary artery disease (REAL-CAD): a randomized superiority trial. Circulation. (2018) 137(19):1997–2009. 10.1161/circulationaha.117.032615 29735587 PMC5959207

[45] Giugliano RP Cannon CP Blazing MA Nicolau JC Corbalán R Špinar J Benefit of adding ezetimibe to statin therapy on cardiovascular outcomes and safety in patients with versus without diabetes mellitus: results from IMPROVE-IT (Improved Reduction of Outcomes: Vytorin Efficacy International Trial). Circulation. (2018) 137(15):1571–82. 10.1161/circulationaha.117.030950 29263150

[46] Kitagawa K Hosomi N Nagai Y Kagimura T Ohtsuki T Maruyama H Cumulative effects of LDL cholesterol and CRP levels on recurrent stroke and TIA. J Atheroscler Thromb. (2019) 26(5):432–41. 10.5551/jat.45989 30318492 PMC6514170

[47] Bohula EA Wiviott SD Giugliano RP Blazing MA Park JG Murphy SA Prevention of stroke with the addition of ezetimibe to statin therapy in patients with acute coronary syndrome in IMPROVE-IT (Improved Reduction of Outcomes: Vytorin Efficacy International Trial). Circulation. (2017) 136(25):2440–50. 10.1161/circulationaha.117.029095 28972004

[48] Amarenco P Kim JS Labreuche J Charles H Giroud M Lee BC Benefit of targeting a LDL (low-density lipoprotein) cholesterol <70 mg/dl during 5 years after ischemic stroke. Stroke. (2020) 51(4):1231–9. 10.1161/strokeaha.119.028718 32078484

[49] Kumbhani DJ Steg PG Cannon CP Eagle KA Smith SC Jr Goto S Statin therapy and long-term adverse limb outcomes in patients with peripheral artery disease: insights from the REACH registry. Eur Heart J. (2014) 35(41):2864–72. 10.1093/eurheartj/ehu080 24585266 PMC4216432

[50] Stavroulakis K Borowski M Torsello G Bisdas T. Association between statin therapy and amputation-free survival in patients with critical limb ischemia in the CRITISCH registry. J Vasc Surg. (2017) 66(5):1534–42. 10.1016/j.jvs.2017.05.115 28807382

[51] Ramos R García-Gil M Comas-Cufí M Quesada M Marrugat J Elosua R Statins for prevention of cardiovascular events in a low-risk population with low ankle brachial index. J Am Coll Cardiol. (2016) 67(6):630–40. 10.1016/j.jacc.2015.11.052 26868687

[52] Tomoi Y Soga Y Iida O Hirano K Suzuki K Kawasaki D Efficacy of statin treatment after endovascular therapy for isolated below-the-knee disease in patients with critical limb ischemia. Cardiovasc Interv Ther. (2013) 28(4):374–82. 10.1007/s12928-013-0188-6 23756936

[53] Dopheide JF Papac L Schindewolf M Baumgartner I Drexel H. Poor attainment of lipid targets in patients with symptomatic peripheral artery disease. J Clin Lipidol. (2018) 12(3):711–7. 10.1016/j.jacl.2018.02.013 29574071

[54] Ridker PM. Residual inflammatory risk: addressing the obverse side of the atherosclerosis prevention coin. Eur Heart J. (2016) 37(22):1720–2. 10.1093/eurheartj/ehw024 26908943

[55] Wang L Liu L Zhao Y Chu M Teng J. Lipoprotein(a) and residual vascular risk in statin-treated patients with first acute ischemic stroke: a prospective cohort study. Front Neurol. (2022) 13:1004264. 10.3389/fneur.2022.1004264 36408516 PMC9671150

[56] Miller M Stone NJ Ballantyne C Bittner V Criqui MH Ginsberg HN Triglycerides and cardiovascular disease: a scientific statement from the American Heart Association. Circulation. (2011) 123(20):2292–333. 10.1161/CIR.0b013e3182160726 21502576

[57] Jorgensen AB Frikke-Schmidt R West AS Grande P Nordestgaard BG Tybjaerg-Hansen A. Genetically elevated non-fasting triglycerides and calculated remnant cholesterol as causal risk factors for myocardial infarction. Eur Heart J. (2013) 34(24):1826–33. 10.1093/eurheartj/ehs431 23248205

[58] Varbo A Benn M Tybjaerg-Hansen A Jorgensen AB Frikke-Schmidt R Nordestgaard BG. Remnant cholesterol as a causal risk factor for ischemic heart disease. J Am Coll Cardiol. (2013) 61(4):427–36. 10.1016/j.jacc.2012.08.1026 23265341

[59] Budoff M. Triglycerides and triglyceride-rich lipoproteins in the causal pathway of cardiovascular disease. Am J Cardiol. (2016) 118(1):138–45. 10.1016/j.amjcard.2016.04.004 27184174

[60] Kromhout D Giltay EJ Geleijnse JM. n-3 fatty acids and cardiovascular events after myocardial infarction. N Engl J Med. (2010) 363(21):2015–26. 10.1056/NEJMoa1003603 20929341

[61] The Risk and Prevention Study Collaborative Group. n-3 fatty acids in patients with multiple cardiovascular risk factors: the Risk and Prevention Study Collaborative Group. N Engl J Med. (2013) 368:1800–8. 10.1056/NEJMoa1205409 23656645

[62] The ACCORD Study Group, Ginsberg HN Elam MB Lovato LC Crouse JR III Leiter LA Linz P Effects of combination lipid therapy in type 2 diabetes mellitus. N Engl J Med. (2010) 362(17):1563–74. 10.1056/NEJMoa1001282 20228404 PMC2879499

[63] The AIM-HIGH Investigators, Boden WE Probstfield JL Anderson T Chaitman BR Desvignes-Nickens P Koprowicz K Niacin in patients with low HDL cholesterol levels receiving intensive statin therapy. N Engl J Med. (2011) 365(24):2255–67. 10.1056/NEJMoa1107579 22085343

[64] Yokoyama M Origasa H Matsuzaki M Matsuzawa Y Saito Y Ishikawa Y Effects of eicosapentaenoic acid on major coronary events in hypercholesterolaemic patients (JELIS): a randomised open-label, blinded endpoint analysis. Lancet. (2007) 369(9567):1090–8. 10.1016/S0140-6736(07)60527-3 17398308

[65] Watanabe T Ando K Daidoji H Otaki Y Sugawara S Matsui M A randomized controlled trial of eicosapentaenoic acid in patients with coronary heart disease on statins. J Cardiol. (2017) 70(6):537–44. 10.1016/j.jjcc.2017.07.007 28863874

[66] Budoff MJ Bhatt DL Kinninger A Lakshmanan S Muhlestein JB Le VT Effect of icosapent ethyl on progression of coronary atherosclerosis in patients with elevated triglycerides on statin therapy: final results of the EVAPORATE trial. Eur Heart J. (2020) 41(40):3925–32. 10.1093/eurheartj/ehaa652 32860032 PMC7654934

[67] Nosaka K Miyoshi T Iwamoto M Kajiya M Okawa K Tsukuda S Early initiation of eicosapentaenoic acid and statin treatment is associated with better clinical outcomes than statin alone in patients with acute coronary syndromes: 1-year outcomes of a randomized controlled study. Int J Cardiol. (2017) 228:173–9. 10.1016/j.ijcard.2016.11.105 27865182

[68] Daida H Nishizaki Y Iwata H Inoue T Hirayama A Kimura K Randomized trial for evaluation in secondary prevention efficacy of combination therapy - statin and eicosapentaenoic acid (RESPECT-EPA) [oral presentation]. Annual scientific sessions of the American Heart Association. November 5–7, 2022, Chicago, IL.

[69] Nelson JR Budoff MJ Wani OR Le V Patel DK Nelson A EPA’s pleiotropic mechanisms of action: a narrative review. Postgrad Med. (2021) 133(6):651–64. 10.1080/00325481.2021.1921491 33900135

[70] Bang HO Dyerberg J. Plasma lipids and lipoproteins in Greenlandic west coast Eskimos. Acta Med Scand. (1972) 192(1–2):85–94. 10.1111/j.0954-6820.1972.tb04782.x 5052396

[71] Bang HO Dyerberg J. Lipid metabolism and ischemic heart disease in Greenland Eskimos. In: Draper HH, editors. Advances in Nutritional Research. Boston, MA: Springer (1980). p. 1–22.

[72] Hirai A Hamazaki T Terano T Nishikawa T Tamura Y Kamugai A Eicosapentaenoic acid and platelet function in Japanese. Lancet. (1980) 2(8204):1132–3. 10.1016/s0140-6736(80)92558-1 6107739

[73] Vascepa [package insert]. Bridgewater, NJ: Amarin Pharma Inc. (2021).

[74] Curfman G. Do omega-3 fatty acids benefit health? JAMA. (2020) 324(22):2280–1. 10.1001/jama.2020.22898 33190151

[75] Food and Drug Administration. FDA briefing document endocrinologic and metabolic drugs advisory committee meeting (2019). Available online at: https://pink.citeline.com/-/media/supporting-documents/pink-sheet/2019/11/vascepa_ac_fda_brfg.pdf?la=en&hash=565284EEEB7DFC84D3CA2B81C9A9A32FD356F656 (Accessed December 20, 2023).

[76] Committee for Medicinal Products for Human Use. Assessment Report: Vazkepa. Amsterdam, The Netherlands: European Medicines Agency (2021).

[77] Orringer CE Jacobson TA Maki KC. National Lipid Association scientific statement on the use of icosapent ethyl in statin-treated patients with elevated triglycerides and high or very-high ASCVD risk. J Clin Lipidol. (2019) 13(6):860–72. 10.1016/j.jacl.2019.10.014 31787586

[78] Kimura K Kimura T Ishihara M Nakagawa Y Nakao K Miyauchi K JCS 2018 guideline on diagnosis and treatment of acute coronary syndrome. Circ J. (2019) 83(5):1085–196. 10.1253/circj.CJ-19-0133 30930428

[79] Cosentino F Grant PJ Aboyans V Bailey CJ Ceriello A Delgado V 2019 ESC guidelines on diabetes, pre-diabetes, and cardiovascular diseases developed in collaboration with the EASD: the task force for diabetes, pre-diabetes, and cardiovascular diseases of the European Society of Cardiology (ESC) and the European Association for the Study of Diabetes (EASD). Eur Heart J. (2020) 41(2):255–323. 10.1093/eurheartj/ehz486 31497854

[80] Miller M Tokgozoglu L Parhofer KG Handelsman Y Leiter LA Landmesser U Icosapent ethyl for reduction of persistent cardiovascular risk: a critical review of major medical society guidelines and statements. Expert Rev Cardiovasc Ther. (2022) 20(8):609–25. 10.1080/14779072.2022.2103541 35876118

[81] Cardoso R Abovich A Boden WE Arbab-Zadeh A Blankstein R Blumenthal RS. The 2021 AHA/ACC/SCAI coronary artery revascularization recommendations: need for emphasis on prevention and future considerations. JACC Adv. (2022) 1(1):100006. 10.1016/j.jacadv.2022.100006 PMC11198690 38939089

[82] Eikelboom JW Connolly SJ Bosch J Dagenais GR Hart RG Shestakovska O Rivaroxaban with or without aspirin in stable cardiovascular disease. N Engl J Med. (2017) 377(14):1319–30. 10.1056/NEJMoa1709118 28844192

[83] Visseren FLJ Mach F Smulders YM Carballo D Koskinas KC Bäck M 2021 ESC guidelines on cardiovascular disease prevention in clinical practice. Eur Heart J. (2021) 42(34):3227–337. 10.1093/eurheartj/ehab484 34458905

[84] Virani SS Newby LK Arnold SV Bittner V Brewer LC Demeter SH 2023 AHA/ACC/ACCP/ASPC/NLA/PCNA guideline for the management of patients with chronic coronary disease: a report of the AmericanHeart Association/American College of Cardiology Joint Committee on Clinical Practice Guidelines. Circulation. (2023) 148(9):e9–119. 10.1161/cir.0000000000001168 37471501

[85] Zelniker TA Wiviott SD Raz I Im K Goodrich EL Bonaca MP SGLT2 inhibitors for primary and secondary prevention of cardiovascular and renal outcomes in type 2 diabetes: a systematic review and meta-analysis of cardiovascular outcome trials. Lancet. (2019) 393(10166):31–9. 10.1016/s0140-6736(18)32590-x 30424892

[86] Kristensen SL Rørth R Jhund PS Docherty KF Sattar N Preiss D Cardiovascular, mortality, and kidney outcomes with GLP-1 receptor agonists in patients with type 2 diabetes: a systematic review and meta-analysis of cardiovascular outcome trials. Lancet Diabetes Endocrinol. (2019) 7(10):776–85. 10.1016/s2213-8587(19)30249-9 31422062

[87] Reyes-Soffer G Ginsberg HN Berglund L Duell PB Heffron SP Kamstrup PR Lipoprotein(a): a genetically determined, causal, and prevalent risk factor for atherosclerotic cardiovascular disease: a scientific statement from the American Heart Association. Arterioscler Thromb Vasc Biol. (2022) 42(1):e48–60. 10.1161/atv.0000000000000147 34647487 PMC9989949

[88] ClinicalTrials.gov. Assessing the impact of lipoprotein (a) lowering with pelacarsen (TQJ230) on major cardiovascular events in patients with CVD (Lp(a)HORIZON) NCT04023552 (2023). Available online at: https://clinicaltrials.gov/ct2/show/NCT04023552 (Accessed December 13, 2023).

